# Selections

**Published:** 1902-01

**Authors:** 


### SELECTIONS.

### CALVES AND PASTEURIZED MILK.

DAIRY farmers will be interested in reading the following letter from Mr. P. Quirk, the manager of the Government Stud Farm at Berry, on the effect of Pasteurized milk in calf-rearing. He says : It has been freely stated that we cannot rear calves on Pasteurized milk at the Government Stud Farm. This I give a flat denial to. All the calves reared at our former farm, Kirkham, were fed on Pasteurized separated milk, and we never lost one of them, and they were the best reared lot I ever saw. Again, at Berry, when we arrived here, our Pasteurizing plant was not erected for some time, so we fed on raw whole milk, with the result that we lost several very valuable calves with white scours. We were at a loss to know what to do, and got veterinary advice and medicine, all to no purpose. Our next move was to erect the Pasteurizing plant and to feed upon Pasteurized milk. Since then we have lost only one calf, and his constitution was undermined by the feeding on raw whole milk.

When pure-bred stock, which are so much harder to rear, can be brought up strong and well developed on Pasteurized milk, surely cross-bred can. One of the main reasons why so many calves die is because the milk is not given at the proper temperature, which should be about blood heat—not hot at one feed and cold at another, as is too often the case. I know thousands of calves that were fed on separated un-Pasteurized milk, and a very high percentage of them died from white scours. One large dairyman offered me £100 if I could give him a recipe to cure white scours, which is contagious. My opinion why such a high percentage of calves is lost is that nature ordained that a calf should suck his food, and not gulp it down, to remain in its stomach an unyielding and indigestible mass which turns to white scours. The glands of the mouth contain the saliva which should pass with the food into the stomach. When the calf is forced to drink its milk out of a bucket these glands are inactive, the saliva does not rise with the food to assist digestion, the animal suffers, becomes bloated and stunted, is frequently scoured, and under these conditions the farmer cannot rear healthy, profitable calves. To overcome these difficulties, we are now using Clarke's patent feeders, with good results, and I am convinced that the salivation excited by the feeders has a nutritious and beneficial effect, and that the old system of "poddying" calves was as cruel to them as it was thriftless and unprofitable to the owner.

Mr. P. H. Morton also says : My attention having been called to a statement made by Mr. Alexander Campbell, M.P., at a meeting of dairy farmers at Kiama a short time ago, to the effect that I had discontinued giving Pasteurized milk to calves on my dairy farm at Barrengarry, I desire to say that such is not the case. All milk, whether for separation, for cream, or for calves, is put through a Pasteurizer. The calves for some time past and now being reared at Barrengarry on Pasteurized skimmed milk (younger ones get some fresh milk added to the Pasteurized) are doing splendidly.—Sydney Station Farm and Dairy, *Agricultural Journal*, October 24, 1901.

---

### A JOKE IN THE WAR OFFICE.

Not often is laughter heard in the solemn office in Pall Mall. But Captain Champion, of the Twenty-first Lancers, and brother to Mr. H. H. Champion, the well-known Socialist and lecturer, has recently provoked even the staidest official to unwonted merriment. This young officer, who, by the by, greatly distinguished himself at Omdurman, was sent a few weeks ago on special remount duty to South Africa, and it is his formal reports home upon his duties which have so gladdened the hearts of the wearied War Office authorities. Captain Champion came to loggerheads with a veterinary surgeon on board the ship. Here is the passage of his report which deals with the unfortunate vet.'s methods in taking the temperature of the horses:

"I didn't at first deem my own knowledge of veterinary science sufficient to suspend him, although his methods were those I had not seen practised before. For instance, putting a thermometer in the horse's mouth and leaving it there. This may be the correct way of taking a fevered animal's temperature, but never having seen it done in this manner it caused a certain amount of prejudice in my mind, to say nothing of the fact that the supply of thermometers under such circumstances naturally proved wholly inadequate, the two which had been sent in the medicine chest being speedily consumed by the aforesaid animals. I, therefore, considered that Mr. ———, though the driver of quite a successful cat and dog practice in ———, was not competent for the treatment of sick horses on board one of H. M. transports."—*M. A. P., Veterinary Record.*

### EXCHANGE.

A Japanese Dairy Company.—Japan is not a dairy country, but there are a few students of dairying who, having obtained an insight into the milk question in this country, have gone back to sow the seed of sanitary milk production. The following circular shows that while they may be weak in English they are strong on good food and clean milk.

### NOTICE.

Kashiyama Milk & Co.

Karuizawa.

The above company, being hither largeing engaged for the milkage business, have already obtained the esteem of the general public for the fresh and pure milk they furnish, and now they are able to extend their business still further as the under mentioned. Any order will be welcomed by the company at a most favorable terms.

1. Ten or more Jersey cows now in hand. Each one is certified by the veterinarian proving her sound health.
2. The pasture land is located in a high and extensive field (Tachikobara) at Karuizawa, where the fresh air and best herbage existed there, keep the cows, without a doubt, much more healthiness.
3. The food provided for them are wheat, hay, and others. We take a special attention for their food, which is, in other words, the material of milk.
4. The fresh and pure milk thus obtained is promptly delivered to the customers twice a day (noon and before noon), and no delay to be occurred.

*Certificate.*

This to certify that the cows for milkage in the possession of Kashiyama Milk Co. are quite healthy and have no diagnosis to be blamed at all. The Veterinarian :

Masanori Shirai.

To Kashiyama Milk & Co., Karuizawa.

—*Hoard's Dairyman*, September 27, 1901.

